# Psychometric Properties of a Chatbot Version of the PHQ-9 With Adults and Older Adults

**DOI:** 10.3389/fdgth.2021.645805

**Published:** 2021-04-30

**Authors:** Gilly Dosovitsky, Erick Kim, Eduardo L. Bunge

**Affiliations:** Psychology Department, Palo Alto University, Palo Alto, CA, United States

**Keywords:** chatbot, assessment, PHQ-9, mobile health, depression

## Abstract

**Background:** The Patient Health Questionnaire-9 (PHQ-9) is a brief depression measure that has been validated. A chatbot version of the PHQ-9 would allow the assessment of depressive symptoms remotely, at a large scale and low cost.

**Objective:** The current study aims to: Assess the feasibility of administering the PHQ-9 in a sample of adults and older adults *via* chatbot, report the psychometric properties of and identify the relationship between demographic variables and PHQ-9 total scores.

**Methods:** A sample of 3,902 adults and older adults in the US and Canada were recruited through Facebook from August 2019 to February 2020 to complete the PHQ-9 using a chatbot.

**Results:** A total of 3,895 (99.82%) completed the PHQ-9 successfully. The internal consistency of the PHQ-9 was 0.896 (*p* < 0.05). A one factor structure was found to have good model fit [*X*^2^ (27, *N* = 1,948) = 365.396, *p* < 0.001; RMSEA = 0.080 (90% CI: 0.073, 0.088); CFI and TLI were 0.925 and 0.900, respectively, and SRMR was 0.039]. All of the demographic characteristics in this study were found to significantly predict PHQ-9 total score, however; their effect was negligible to weak.

**Conclusions:** There was a large sample of adults and older adults were open to completing assessments *via* chatbot including those over 75. The psychometric properties of the chatbot version of the PHQ-9 provide initial support to the utilization of this assessment method.

## Introduction

Depressive disorders affect more than 264 million people of all ages globally according to the World Health Organization (WHO) (1). The prevalence rate of depressive disorders for adults and older adults around the world varies from 10 to 20% (2). With the high prevalence rates of depression, developing scalable and low-cost instruments to assess depression in adults and older adults is an essential public health need. Many individuals with depression do not access assessment (or treatment) due to stigma (3), economic barriers (4), or lack of engagement with specialty mental health services and high engagement with primary care where depression is not always screened (5). The Patient Health Questionnaire-9 (PHQ-9) (6) is a brief measure of depression that has been extensively validated (7–10). A chatbot version of the PHQ-9 would allow the assessment of depressive symptoms interactively, remotely, at a large scale, and low cost, however, no studies to date have assessed the validity of a chatbot version of the PHQ-9. Developing large scale and remote means of assessing depression is highly relevant since it would allow individuals to self-assess in an interactive way using just their personal devices. Having means for remote assessment is especially necessary for adults and older adults during the COVID-19 pandemic, since they are at a higher risk of getting infected during in-person visits to health care centers (11). Having the option to complete assessments on personal phones in a more interactive way can complement computer delivered assessments to reach a greater number of people.

The PHQ-9 is a reliable measure of depression with a Chronbach's alpha of 0.89 (6). Exploratory factor analyses of the PHQ-9 have shown evidence for both a one factor and two factor structure. Dum et al. (12) found that the PHQ-9 had a one-factor structure where all items had a factor loading over 0.35 and this factor accounted for 59.97% of the variance. Two other studies found that the PHQ-9 was best explained by one overarching factor; a study with racially and ethnically diverse primary care patients (8), and another study with a general population of adults in Hong Kong (9). Exploratory factor analyses of the PHQ-9 have identified a two-factor model (10). In a study on the PHQ-9 in adults and older adults in Taiwan, a two-factor structure of somatic and non-somatic symptoms was found (13), and Beard et al. (14) also found a two-factor structure, one consisting of affective and cognitive items and another consisting of somatic items.

Several studies have shown that the PHQ-9 can reliably be used in different formats such as computers (7, 15), smartphone apps (16), and tablets (17). Erbe et al. (7) found a strong correlation (0.92) between a computerized version of the PHQ-9 and the paper and pencil version at an inpatient routine mental health clinic in Germany. Fann et al. (15) assessed the feasibility and validity of administering the PHQ-9 to adult oncology and related specialty patients using a touch screen computer (15). Although, Fann et al. (15) found that the PHQ-9 administered through a touch screen computer provided valid data, the sample was relatively affluent and educated, so comfortability with technology may be lower in other samples. BinDhim et al. (16) assessed the PHQ-9 administered using a smartphone app with a sample of 8,241 adults, with a mean age of 29.4 years old, and found a completion rate of 73.9%. While this study shows that a large number of participants were willing to share their personal information through this anonymous smartphone app it is not clear if this is generalizable to older adults. Spangenberg et al. (17) assessed interformat reliability between a tablet and a paper-and-pencil version of the PHQ-9 with a sample of elderly primary care patients and concluded that the tablet version of the PHQ-9 was a valid way to electronically assess depressive symptoms in elderly patients.

Overall, the findings from the digital versions of the PHQ-9 (computer, tablet, and smartphone app) are congruent with the findings of Coons et al. (18) that show that electronic-based patient-reported outcomes adapted from a paper-based format produce equivalent or even more reliable data than paper versions. Additionally, a meta-analysis by Weigold et al. (19) found that participants are significantly more likely to complete and return assessments than in computerized versions. While the mentioned studies provide support for the utilization of digital versions of the PHQ-9, none of these studies focused on chatbots and older adults. Administration *via* chatbot has two distinct features to an online delivery. The first, is that with a chatbot, administration is interactive and can be triggered by the chatbot whereas online delivery requires the user to initiate the interaction. Second, it may be completed through text messaging, which presents each question one at a time in a more animated format rather than the typical, static questionnaire. These differences may make the chatbot version more convenient than the online version, however there are no studies that currently assess the validity of the PHQ-9 delivered by a chatbot.

In recent years, there has been an increased interest in the integration of chatbots for assessment (20) and treatment of depression (21–23). While Fitzpatrick et al. (22) and Fulmer et al. (23) reported improvements in depressive symptoms using an interactive version of the PHQ-9 through a chatbot, the psychometrics properties have not been reported. To our knowledge, only two studies reported psychometric properties of chatbot assessments. One study on the PHQ-9 delivered in Spanish by a chatbot found that the psychometric properties are comparable to the paper and pencil version (24). Another study of a loneliness scale that found quantitative equivalence between the chatbot and paper and pencil version (20). Overall, no studies to date have focused on the assessment of the PHQ-9, in English, *via* chatbot, for adults and older adults. Since prevalence of depression in adults and older adults is 10–20% (2), scalable methods of assessing depression for this population are needed. Thus, the current study aims to: (1) Assess the feasibility of administering the PHQ-9 in a sample of adults and older adults *via* chatbot. (2) Assess the psychometric properties (factor analysis and reliability) of the PHQ-9 when delivered *via* a chatbot to a general sample of adults and older adults. (3) Identify the relationship between demographic variables, including sex, age, ethnicity, living status, education level, and employment status, and PHQ-9 total scores.

## Method

### Participants

The participants of this study were 3,895 adults and older adults (over 65 years of age) in the US and Canada who were recruited through Facebook from August 2019 to February 2020, for a study on the efficacy of a chatbot for social isolation in adults and older adults reported elsewhere.

### Materials

The materials for this study was the chatbot (Tess) version of the PHQ-9. Tess is a mental health chatbot that uses an AI-based computer program to engage with users to teach coping skills and provide support. Users can chat with Tess through text message conversations or Facebook Messenger. Other studies provide more information on how Tess works (21).

The PHQ-9 includes questions on the nine criteria of major depressive disorder. Scores on the PHQ-9 range from 0 to 27, each of the items scored from a 0 to 3 where 0 represents the symptom is not present at all and a 3 represents the symptom is present nearly every day. PHQ-9 scores can be categorized into the five severity levels of the PHQ-9 where a total score of <5 is “minimal,” a score of 5–9 is “mild,” a score of 10–14 is “moderate,” a score of 15–19 is “moderately severe,” and a score of 20–27 is “severe” (6). The chatbot version of the PHQ-9 would initially provide two messages to prepare participants to take the screener: “Please answer the questions as they apply to your experience over the past 2 weeks. You can respond with 0, 1, 2, or 3,” and “0 Means not at all affected, 1 means affected for several days, 2 means affected for more than half the days, and 3 means affected nearly every day. Make sense?” If users did not respond affirmatively to instructions, they were delivered in a different way until the user said they understood. Users were then asked each question on the PHQ-9 in the following format: “Over the last 2 weeks, how often have you been bothered by the problem of feeling little interest or pleasure in doing things? 0 = not at all, 1 = several days, 2 = more than half days, 3 = nearly everyday.” Users were reminded of the meaning of each number of the scale in every other question. To each question users were expected to respond with a number 0 to 3.

### Procedures

Recruitment efforts for this study primarily focused on Facebook advertisements. Facebook ads used text focusing on depression, loneliness, and social isolation to ensure those who clicked on the advertisement would find the content relevant to their needs. Some participants may have also been referred to the study from participants already enrolled. Users expressed their interests by initiating the conversation with the chatbot *via* Facebook Messenger as they were prompted to do in the advertisements.

After users expressed an interest in the chatbot, they were sent an introductory message through Facebook Messenger which explained what a chatbot is and included a link to the chatbot's privacy policy and a consent form. To access these forms, users needed to click on the links which directed users out of Facebook messenger. If participants agreed, they were directed back to the Facebook Messenger conversation to begin the assessment process. Following these messages, users received information on what they should do if they are experiencing a crisis while using the chatbot (Tess). Participants were then asked a series of demographic questions and completed the following assessments: Duke Social Support Index, Friendship Scale, Loneliness Scale, Confidence Scale, and the PHQ-9. For this study, only the participant's responses to the PHQ-9 are evaluated. Next, participants were allowed to chat with Tess for ~6 months. After completing an intervention, participants were asked about the usefulness of the chatbot Tess and were given the opportunity to complete the PHQ-9 again. This study was determined to be non-human subjects by the Institutional Review Board at Palo Alto University (Assurance Number: FWA00010885).

### Analysis

Frequency distributions for demographic characteristics and completion rates for PHQ-9 scores were conducted using R (20). The internal consistency of the PHQ-9 was assessed using Cronbach's alpha and inter-item correlations using Spearman's rank correlation coefficients. Both Bartlett's test of sphericity and Kaiser-Meyer-Olkin measure of sampling adequacy were used to determine suitability of factor analysis.

The factor structure of the PHQ-9 was examined by conducting a split-half exploratory factor analysis and confirmatory factor analysis using MPlus Version 8.3 (21). An exploratory factor analysis using maximum likelihood estimation with geomin oblique rotation was conducted. Scree plots, eigenvalues (≥1), factor loadings (>0.4), and model fit of one and two factor structures were found. Then, a confirmatory factor analysis was conducted to evaluate model fit. To determine model fit, chi-squared model fit statistics (*X*^2^), comparative fit index (CFI), Tucker-Lewis index (TLI), the room mean square error of approximation (RMSEA) with 90% confidence intervals, and standardized root mean square residual (SRMR) were found. Kline (25) and Hooper et al. (26) criteria for goodness of fit were used. The following criteria were used to determine goodness of fit: (1) CFI ≥0.9; (2) TLI ≥0.95; (3) RMSEA <0.08; (4) SRMR <0.08; (5) goodness of fit *p* > 0.05.

## Results

### Demographics

There were 3,902 participants who provided consent. Seven participants' presented errors in completion of the PHQ-9 (participants submitted a response that was out of range) and their scores were excluded from the analyses. For the 3,895 participants who completed correctly, the PHQ-9 demographic characteristics are in Table 1. The majority of participants identified as female (*N* = 3,575, 91.79% of sample). The largest age group was participants who reported being 55–60 years old (*N* = 1,489, 38.22%). Most participants identified as White (*N* = 2,336, 59.97%), and were not living alone (*N* = 2,675, 68.68%). The largest number of participants reported that the highest level of education achieved was high school diploma or equivalent (*N* = 1,648, 42.31%). Regarding employment status, most participants reported being unable to work (*N* = 1,587, 40.74%). The average total PHQ-9 score was 17.60 with a standard deviation of 6.90 points.

**Table 1:** Demographic characteristics (total *N* = 3,895).

	***N* (%)**	**Mean PHQ-9 total score**	**Median**	**Minimum**	**Maximum**	***SD* of PHQ-9 total score**	**IQR (Q1–Q3)**
Gender							
Female	**3,575 (91.79)**	17.76	19	0	27	6.821	(13–23)
Male	256 (6.57)	15.83	17	0	27	7.581	(11–22)
Other	12 (0.31)	17.83	19.50	7	27	6.408	(11.25–22.75)
Decline to state	52 (1.33)	15.25	17	0	27	7.685	(8.25–21.75)
Age							
Under 55	1,256 (32.24)	18.76	20	0	27	6.574	(15–24)
55–60	**1,488 (38.22)**	17.68	19	0	27	6.702	(13–23)
61–65	624 (16.02)	16.90	18	0	27	7.148	(13–23)
66–70	272 (6.98)	16.19	18	0	27	7.045	(11.25–21.75)
71–75	100 (2.57)	14.36	15.5	0	27	7.473	(8–20)
76–80	41 (1.05)	13.78	13	0	27	6.839	(8.5–19.5)
80 and older	12 (0.31)	10.83	10.5	0	27	8.664	(1.5–17.75)
Decline to state	102 (2.62)	15.53	16	0	27	7.634	(9–22)
Ethnicity							
Asian	86 (2.2)	16.53	18	0	27	7.617	(12–23)
Black	358 (9.2)	14.65	16	0	27	7.642	(8–21)
White	**1,879 (48.2)**	18.13	20	0	27	6.577	(14–23)
Latin American	81 (2.1)	18.38	20	0	27	6.914	(14–24)
Indigenous	123 (3.2)	17.72	20	0	27	6.827	(14–23)
Mixed	7 (0.2)	18	18	7	26	6.733	(14.5–23)
Other	103 (2.6)	16.24	18	0	27	7.413	(11–23)
Decline to state	1,258 (32.3)	17.76	19	0	27	6.151	(13–23)
Living status							
Living alone	1,155 (29.65)	17.30	19	0	27	7.100	(13–23)
Not living alone	**2,675 (68.68)**	17.78	19	0	27	6.779	(14–23)
Decline to state	65 (1.67)	15.20	16	0	27	7.888	(8.5–21)
Education							
Schooling but not a HS diploma	890 (22.85)	18.17	20	0	27	6.783	(14–23)
HS diploma/equivalent	**1,648 (42.31)**	17.57	19	0	27	6.937	(13–23)
College certificate or diploma, trade, vocational or technical school, CEGEP	842 (21.62)	17.56	19	0	27	6.732	(13–23)
University	164 (4.21)	15.57	16	0	27	7.325	(11–21.75)
PhD or equivalent	20 (0.51)	14.00	17	0	26	8.272	(6.75–20)
Other	331 (8.50)	17.51	19	0	27	6.97	(14–23)
Employment status							
Employed, full time	484 (12.43)	16.29	17	0	27	6.929	(11–22)
Employed, part time	311 (7.98)	16.73	18	0	27	6.853	(12–22)
Unemployed and currently looking for work	335 (8.60)	18.55	20	0	27	6.809	(15–24)
Unemployed and not currently looking for work	194 (4.98)	18.13	20	0	27	7.004	(14–24)
Student	27 (0.69)	17.30	19	0	26	7.660	(10–24)
Retired	589 (15.12)	15.75	17	0	27	7.334	(10.5–22)
Volunteer	20 (0.51)	15.60	16.5	0	27	9.316	(9–24)
Homemaker	182 (4.67)	16.55	18	0	27	7.329	(11–23)
Self-employed	118 (3.03)	16.30	17	0	27	7.044	(11–22.25)
Unable to work	**1,587 (40.74)**	18.83	20	0	27	6.353	(15–24)
Decline to state	48 (1.23)	17.69	18	0	27	6.941	(14–23.75)

*Bolded means in the table represent the subgroups with the highest mean score*.

### Feasibility and Completion

Out of the 3,902 participants, 3,895 (99.82%) completed the PHQ-9 successfully. The seven participants that did not complete the PHQ-9 correctly identified as female. One of these participants was under 55 years old, two were between the ages of 55 and 60, two were between the ages of 66 and 70, and one was between 71 and 75. Four of the participants identified as White, one identified as Latin American, one reported they did not know, and one declined to state.

### Psychometric Properties

The internal consistency of the PHQ-9 was found using Chronbach's alpha. Based on the 3,895 participants who responded to the 9 questions of the PHQ-9, Chronbach's alpha was 0.896 (*p* < 0.05). The inter-item correlations ranged from 0.25 to 0.59 using Spearman's rank correlation to account for non-normality of the PHQ-9. Bartlet's test of sphericity was run to determine that items are suitable for factor analysis (*p* < 0.05). Kaiser-Meyer-Olkin (KMO) measure of sampling adequacy was 0.904 indicating good sampling adequacy.

#### Reliability

Internal reliability calculated by Chronbach's alpha. Based on the 3,895 participants who responded to the nine questions of the PHQ-9, Chronbach's alpha was 0.896 (*p* < 0.05).

#### Factor Analysis

To identify and validate the factor structure of the PHQ-9, a split-half exploratory factor analysis (EFA)/confirmatory factor analysis (CFA) was conducted using maximum likelihood estimation with geomin oblique rotation. Each participant was randomly assigned to either the EFA (*N* = 1,948) or CFA (*N* = 1,947) group. For the EFA, eigenvalues, factor loadings, and fit indices were evaluated. Only one factor structure yielded an eigenvalue ≥1. All factor loadings for the one factor model had loadings of 0.467 or higher. Factor loadings for the one and two-factor models are presented in Table 2. The one factor structure indicated poor model fit as evidenced by *X*^2^ (27, *N* = 1,948) = 365.396, *p* < 0.001; CFI and TLI were 0.925 and 0.900, respectively, and SRMR was 0.039. RMSEA = 0.080 (90% CI: 0.073, 0.088) indicated good model fit. EFA indicated some support for a two and three factor structure. However, given the low loadings and cross loadings in the two and three factor structure as well as parsimony, a one-factor structure was determined for this sample. Communalities for each item is presented in Table 2. A CFA was conducted for the other half of the sample, demonstrating poor model fit [*X*^2^ (27, *N* = 1,947) = 417.652, *p* < 0.001]; the CFI and TLI were 0.932 and 0.910, respectively, and SRMR was 0.039. RMSEA was 0.086 (90% CI: 0.079, 0.094) and indicated mediocre fit. It should be noted that Bentler and Bonnet (27) found that large sample size can influence goodness of fit in chi-square models.

**Table 2:** Results of exploratory factor analysis.

	**One factor**		**Two factors**	
**PHQ-9 item description**	**Factor loadings**	**Communalities**	**Factor I loadings**	**Factor II loadings**
Feeling down, depressed, hopeless	0.744^*^	0.554	0.389	0.401^*^
Anhedonia	0.573^*^	0.329	0.335	0.276
Poor appetite	0.643^*^	0.414	0.230^*^	0.458^*^
Difficulty sleeping	0.639^*^	0.408	0.735^*^	−0.007
Psychomotor retardation/agitation	0.617^*^	0.380	−0.021	0.676^*^
Little energy	0.677^*^	0.458	0.771^*^	0.006
Feeling bad about yourself	0.740^*^	0.547	0.132	0.646^*^
Trouble concentrating	0.712^*^	0.507	0.002	0.759^*^
Thoughts of suicide/death	0.467^*^	0.218	−0.015	0.499^*^

* *Indicates value is significant at p < 0.05, items with loadings above 0.400 are bolded. Factor analysis was conducted with maximum likelihood estimation extraction with oblique rotation. Communalities were reported for variance explained of each item in the one factor model*.

### Differences in Scores Based on Demographic Variables

To analyze differences caused by demographic characteristics, we examined the distribution of scores. Skew (−0.74) and kurtosis (−0.28) indicated a normal distribution of scores, however; Shapiro-Wilk (W = 0.934, *p* < 0.01) and the Kolmogorov-Smirnov test (D = 0.12, *p* < 0.01) indicated non-normally distributed data. Levene's, Fligner-Killeen, and Brown-Forsythe homogeneity tests indicated non-homogeneity of variance for gender and age. To account for the non-normality and heteroscedasticity of variance, non-parametric testing was conducted for further analyses. PHQ-9 total score was evaluated based on the user's demographic characteristics. Given the non-normal distribution of PHQ-9 scores, Kruskal-Wallis ANOVAs were conducted. ANOVAs for all demographic variables were significant at *p* < 0.05. Results of ANOVA for all demographic characteristics are presented in Table 3 below. For all variables *E*^2^ indicate negligible to weak effect size and that demographic variables were predictive of PHQ-9 total score.

**Table 3:** Results of Kruskal-Wallis ANOVA examining PHQ-9 total score across demographic variables.

	**Kruskal-Wallis *X^**2**^***	***p*-value**	***E^**2**^***	**Interpretation of effect size**
Gender	20.45	<0.01	0.00525	Negligible
Age	106.46	<0.01	0.0273	Weak
Ethnicity	66.963	<0.01	0.0172	Weak
Living status	9.0161	<0.01	0.00232	Negligible
Education	29.618	<0.01	0.00761	Negligible
Employment status	132.67	<0.01	0.0341	Weak

*E^2^ represents one measure of the effect size of the Kruskal-Wallis ANOVA. Effect sizes 0.00 < 0.01 were considered negligible and those 0.01 < 0.04 were considered weak*.

#### Severity

Since PHQ-9 scores were not normally distributed, breakdown by severity was assessed. The breakdown of participants by severity levels is presented in Table 4. The Severe category had the largest number of participants compared to any other category (*N* = 1,872, 48.06%) with an average score of 23.78, and was the largest category for most age groups.

**Table 4:** Prevalence of severity rates (total *N* = 3,895).

	***N***	**Avg. PHQ-9 total score**	**Under 55**	**55–60**	**61–65**	**66–70**	**71–75**	**76–80**	**80 and older**
Minimal (0–4)	243	2.03	55	82	50	22	15	6	3
Mild (5–9)	335	7.16	84	123	59	31	15	5	2
Moderate (10–14)	540	12.11	143	219	92	41	16	11	2
Moderately severe (15–19)	905	17.16	267	348	160	69	27	9	4
Severe (20–27)	**1,872**	23.78	707	716	267	109	27	10	1

*Bolded means in the table represent the subgroups with the highest mean score*.

## Discussion

The current study analyzed data from 3,895 adults and older adults who completed the PHQ-9. The majority of which were female, over 55, and around 40% of the sample identified as an ethnicity other than White. Analysis of the psychometric properties of the chatbot version of the PHQ-9 are consistent with psychometrics of the paper and pencil version. A one structure model was found to have good model fit with strong internal consistency. All demographic characteristics analyzed in this study had a significant, but weak effect on the PHQ-9 total score.

Regarding the feasibility of using a chatbot to assess depressive symptoms, two points should be noted. First, the large sample recruited in this study may indicate that individuals were open to completing assessments *via* chatbot. Additionally, the high completion rate (99.8%) shows that the assessment was relatively easy for participants to complete, including those over 75, indeed, all of the participants over 75 (*N* = 53) completed the chatbot version of the PHQ-9 correctly. To note, a previous study of a smartphone app version of the PHQ-9 showed a lower completion rate of 73.9% (16). These results show that the chatbot attracted a large sample of adults and older adults, with low education, with a large portion of individuals that were unable to work; and nonetheless yielded high completion rates the PHQ-9. Thus, assessments *via* chatbots could be considered as a viable modality for assessment delivery for older adults.

The psychometric properties found provide evidence of the validity of the chatbot version of the PHQ-9. The internal reliability was 0.896 which indicated good reliability between items. The EFA and CFA showed that a one factor structure was most appropriate even though there was some support for two and three factor structures. However, given the support for a one factor structure for ethnically diverse samples in a paper version of the PHQ-9 (8, 9, 12), low loadings or cross loadings in the two and three factor structure, and parsimony, a one-factor structure was determined for this sample. All factor loadings for the one factor model had loadings of 0.467 or higher. The item with the highest factor loading was feeling depressed (0.744) and the lowest was thoughts of death (0.467). Since dysphoria is one of the main symptoms of depression, it was expected that this item would have one of the highest loadings in a single factor structure. Interestingly, Fiske et al. (28) found that older adults (over 65 years of age) are less likely to present symptoms of dysphoria and much more likely to report loss of interest in life and somatic symptoms. It is possible that this difference is explained by the inclusion of adults (under 55) and older adults in this study and that the recruitment ads focused on sadness and loneliness.

There were no to weak differences in scores when analyzed by demographic characteristics. Gender, age, ethnicity, living status, education, and employment status were not strong predictors of PHQ-9 total scores. Though the sample was diverse, the differences in group sizes may be what led to this finding. Future studies should prioritize including a representative sample to understand more about the relationship between demographic characteristics and PHQ-9 total score.

Due to COVID-19, there is a need for digital assessments to address the immediate mental and physical health needs of patients of all ages, especially the older ones that are at a higher risk. Completing a paper and pencil assessment and even exchanging a tablet with a preloaded assessment in the waiting room may increase disease transmission. This study shows that adults and older adults were able to complete the PHQ-9 on their personal device which represents a safer alternative. Additionally, the psychometric properties of the chatbot version of the PHQ-9 support the integration of this assessment in the studies utilizing chatbots to treat depression.

### Limitations

One limitation in this study was that only the chatbot version of the PHQ-9 was administered. Future studies on chatbot administrations of the PHQ-9 should compare reliability between a paper version and chatbot version as was done previously with the computer format of the PHQ-9 (7) and touch screen computer version (15). Additionally, construct validity and criterion validity have not been assessed, thus these findings should be interpreted as preliminary.

Regarding the sample of participants, since the majority were female the results may not generalize to adults of other genders. While over 40% of the sample reported an ethnicity other than White, studies with more diverse samples are needed. Additionally, age was assessed categorically rather than continuously with the youngest age option being “<55.” For the 32.34% of the final sample who indicated they were younger than 55, the variance and lowest age is not known. Thus, future studies should assess age continuously.

Lastly, participants in this study were recruited through Facebook and engaged with the chatbot only through Facebook Messenger. Thus, the results of this study should be interpreted with caution as it may not generalize to older adults who are less technologically savvy. To gain a better understanding of older adults' openness to interacting with the chatbot version of the PHQ-9, recruitment efforts may need to be diversified beyond Facebook.

### Conclusions

Finding a low cost and scalable format to deliver depression screeners would allow large numbers of people to be assessed. The current study showed that the PHQ-9 delivered through a chatbot was reliable and presented a one factor structure with depressed mood as the item with the highest loading. These findings provide preliminary support to the utilization of a chatbot version of the PHQ-9 for the assessment of depression for adults and older adults which represents a safe assessment method during the current pandemic.

## Data Availability Statement

The raw data supporting the conclusions of this article will be made available by the authors, without undue reservation.

## Ethics Statement

The studies involving human participants were reviewed and approved by Palo Alto University. The patients/participants provided their written informed consent to participate in this study.

## Author Contributions

All authors listed have made a substantial, direct and intellectual contribution to the work, and approved it for publication.

## Conflict of Interest

The authors declare that the research was conducted in the absence of any commercial or financial relationships that could be construed as a potential conflict of interest.
